# Networks Disrupted in Linguistic Variants of Frontotemporal Dementia

**DOI:** 10.3389/fneur.2019.00903

**Published:** 2019-08-23

**Authors:** Pablo Alexander Reyes, Andrea del Pilar Rueda, Felipe Uriza, Diana L. Matallana

**Affiliations:** ^1^Radiology Department, Hospital Universitario San Ignacio, Bogotá, Colombia; ^2^Medicine School, Aging Institute, Pontificia Universidad Javeriana, Bogotá, Colombia; ^3^BASPI, Faculty of Engineering, Pontificia Universidad Javeriana, Bogotá, Colombia

**Keywords:** diffusion tensor imaging, primary progressive aphasia, frontotemporal dementia, fractional anisotropy, white matter, structural connectivity

## Abstract

The non-fluent/agrammatic variant of primary progressive aphasia (nfvPPA) and semantic variant (svPPA) of frontotemporal dementia (FTD) are neurodegenerative diseases. Previous works have shown alterations of fractional anisotropy (FA) and mean diffusivity (MD) from diffusion tensor images (DTIs). This manuscript is aimed at using DTI images to build a global tractography and to identify atrophy patterns of white matter in each variant. Twenty patients with svPPA, 20 patients with nfvPPA, 26 patients with behavioral variant of FTD (bvFTD) and, 33 controls were included. An analysis based on the connectivity of structural networks showed changes in FA and MD in svPPA and nfvPPA with respect to bvFTD. Much damage in the internal networks of the left temporal lobe was found in svPPA patients; in contrast, patients with nfvPPA showed atrophy in networks from the basal ganglia to motor and premotor areas. Those findings support the dual stream model of speech and language.

## 1. Introduction

Primary progressive aphasias (PPAs) are clinical syndromes characterized by a progressive and insidious alteration in speech, grammar, or language (1) comprehension. At least two PPAs have been associated with the pathology of frontotemporal lobar degeneration (FTLD): semantic variant of primary progressive aphasia (svPPA) and non-fluent/agrammatic variant (nfvPPA) (2, 3) and logopenic variant (lvPPA), which is associated with Alzheimer's disease (AD) pathology. The classification based on these three types is supported by clinical characteristics of language disturbances and cerebral pattern atrophy (4).

Disturbances in comprehension and semantic knowledge are the major characteristics of svPPA. Hence, the degradation of knowledge generates changes in naming, categorizing, conceptualizing (5, 6), surface dyslexia, and spared speech production (7). Brain atrophy in svPPA was found in temporal lobes bilaterally in anterior and lateral regions. Despite bilateral atrophy, some reports found greater changes in the left temporal than in the right (8). Also, regarding gray matter atrophy, the patients had a reduction in the temporal pole, inferior temporal gyrus, insula, and amygdala in the left hemisphere (9). The semantic impairment has been associated with hypoperfusion in anterior regions of the left temporal lobe in contrast. Additionally, changes in the right hemisphere are related to extending the semantic network (10) and knowledge of self (7).

The nfvPPA include the presence of agrammatism and effort (4) toward spontaneous speech. Some studies have reported other alterations related to language such as dysprosody, phonetic errors, and difficulty with verb naming and comprehension tasks (7). Additionally, there are reports of motors like extra-pyramidal syndrome (11). Atrophy in inferior frontal regions (4, 12) such pars opercularis (7), as well as in the insula, premotor region, supplementary motor area, and striatum was reported in patients with nfvPPA with techniques based on MRI. However, similar regions are reported in functional MRI.

The lvPPA is a variant associated with AD (13). However, some reports mentioned that criteria are not sensitive fo AD neuropathology (14). Clinical characteristics as impaired lexical retrieval or anomia are common speech alterations in both diseases (15). Also, patients with lvPPA show sentence comprehension deficits, regardless of syntactic complexity due to alteration on phonological loop (16). Furthermore, atrophy in parietotemporal junction and has been associated with lvPPA (15).

White matter changes are reported in both nfvPPA and svPPA. The dorsal language pathway between the parietal and frontal was identified in patients with nfvPPA and, according to the progression of the disease in gray matter, the areas underlying those were compromised. Other pathways have been reported, such as in the arcuate, splenium of the corpus callosum, and left uncinate fasciculus (17, 18). In contrast, the pathways with neurodegeneration in svPPA are located in the ventral tracts that connect the temporal lobe with the orbitofrontal and occipital (7). Additionally, the inferior longitudinal fasciculus, anterior callosal fibers, arcuate, and uncinate were severely altered bilaterally (17). The studies have shown similarities and differences between both syndromes and have highlighted the necessity for more studies on white matter. In addition, in most studies, the pattern atrophy in white matter has been based on designs of cases and controls. Few studies have compared variants or PPA with other diseases (19).

The exploration of white matter atrophy can be done from diffusion images by metrics such as fractional anisotropy (FA), mean diffusivity (MD), radial diffusivity (20), and similar. Further, there are no standard approaches to obtaining these metrics as a result of the heterogeneity in acquisition images and protocols of processing and analysis (17). The more common methods are analysis of the whole brain from FA images with tensor-based morphometry (17, 18), the tract-based statistical approach (21), and other techniques based on specific tracts.

Patients with svPPA and nfvPPA exhibit behavioral changes in mild and severe stages. Repetition, compulsiveness, lacking in sympathy and empathy for others, and eating disorders are present in svPPA (22–24). In addition, patients with nfvPPA have similar disturbances such as apathy, aberrant eating, as well as impulsive and obsessive-compulsive behaviors (25, 26). Owing to this overlapping of symptoms, the clinical diagnosis can be compromised not only by the complexity of language evaluation but also by the behavioral changes.

This study is aimed at identifying the degeneration of white matter in terms of structural connectivity at the linguistics variants of FTD. Patients with lvPPA were not included given their probability of AD pathologies. Several metrics from diffusion images will be estimated and a whole structural connectivity with analysis based on the network will be implemented. Finally, comparison groups of svPPA and nfvPPA (in moderate stage with behavior disturbances) with a group of patients with the behavioral variant of FTD and controls to determine the signatures specific in each PPA will be created. We hypothesize that svPPA and nvfPPA patients have specific and disrupted networks between them and with respect to bvFTD and controls.

## 2. Methods

### 2.1. Sample

Sixty-six patients were selected at a memory clinic at the Hospital Universitario San Ignacio: 20 svPPA (mean age 60.3 ±7.65, 20 nfvPPA (mean age 63.63 ±6.87), 26 bvFTD (mean age 64.38 ±5.72) and, 33 healthy controls (mean age 61.3 ±7.22) (see Table 1). Patient fulfilled the Lund and Manchester criteria (27) the revised criteria for probable bvFTD (28) and, criteria for the 3 variants of PPA—nonfluent/agrammatic, semantic, and logopenic (4). All patients had Spanish as their primary language and had an informant. All patients were diagnosed and classified in the memory clinic by a multidisciplinary team focused on neurology, neuropsychology, geriatrics, and psychiatry. Healthy controls were excluded if they had a history of psychiatry or neurological illness, substance abuse, and other causes of brain damage. This study was approved by the ethical committee of the Hospital Universitario San Ignacio/Pontificia Universidad Javeriana and all participants signed the informed consent form.

**Table 1:** Demographic, clinical, and cognitive data from patients with controls, svPPA, nfvPPA, and bvFTD.

**Group**	**svPPA**	**nfvPPA**	**bvFTD**	**Controls**	***Post-hoc* Test**
Number (n)	20	20	26	33	–
Age	60,3 (7.65)	63.63 (6.87)	64.38 (5.72)	61.3 (7.22)	*p* > 0.05
Disease duration (y)	5.85 (3,15)	4.21 (2.57)	6.21 (5.05)	–	*p* > 0.05
Education (y)	12,3 (5.85)	11.62 (6.32)	14.4 (5.13)	14.3 (5.06)	*p* > 0.05
FBI	25.5 (13.6)	23.4 (13.9)	27.9 (12.6)	–	*p* > 0.05
CUSPAD	9.65 (7.73)	5.57 (6.09)	9.71 (6.52)	–	*p* > 0.05
MoCA test	11.9 (6.24)	8.86 (5.75)	19.88 (4.5)	26.3 (2.48)	b,c,d,e,f
RFC	27.89 (9.3)	16.75 (12.72)	21.47 (11.08)	32.8 (4.8)	a,e,f
Proverbs	1.1 (2.3)	2.38 (3.03)	4.1 (3.4)	8.6 (1.99)	b,d,e,f
Digit symbol coding	15.38 (14.42)	18.85 (11.74)	7.89 (5.4)	48.9 (17.6)	b,d,e,f
Language tests					
Semantic Fluency	4.50 (3.59)	5.35 (2.9)	13.13 (4.28)	16.7 (3.39)	b,c,d,e,f
Phonemic Fluency	5 (3.9)	4.69 (3.97)	13.21 (5.12)	15.1 (4.89)	b,c,d,e,f
Naming (% of corrects)	0.89 (0.21)	0.48 (0.29)	0.86 (0.09)	0.95 (0.04)	b,c,d,e
Total words (SS)	233.4 (165.8)	120 (58.9)	206.6 (140.28)	–	a
Repetitions rate (SS)	0.039 (0.017)	0.086 (0.048)	0.028 (0.025)	–	a,c
Pauses rate (SS)	0.064 (0.05)	0.28 (0.08)	0.1 (0.06)	–	a,c

*The numbers are the mean and standard deviation, svPPA, semantic variant PPA; nfvPPA, non-fluent variant; bvFTD, behavioral variant FTD; FIB, Frontal behavioral inventory; CUSPA, Columbia University Scale for Psychopathology in Alzheimer's Disease; RFC, Rey figure copy; SS, spontaneous speech*.

a *p < 0.05 svPPA vs. nfvPPA*;

b *p < 0.05 svPPA vs. bvFTD*;

c *p < 0.05 nfvPPA vs. bvFTD*;

d *p < 0.05 Controls vs. svPPA*;

e *p < 0.05 Controls vs. nfvPPA*;

f *p < 0.05 Controls vs. bvFTD*.

### 2.2. Cognitive and Behavioral Assessment

Patients underwent clinical evaluation, which was administered by a neuropsychologist and neurologist with 10 years' experience in a memory clinic. Cognitive battery was measured including the global cognitive functioning MoCA test (29), language abilities with semantic and phonemic fluency (30), confrontation naming with a naming test for dementia (31), visuospatial abilities with Rey-Osterrieth figure copy (32), verbal comprehension with proverbs (33), speed and memory with digit symbol coding of WAIS (34), and spontaneous speech with the order “Choose a day of your life or a memory, no matter what it is, whether happy or sad, and tell me about it.” The parameters of this evaluation were reported previously (35). The behavioral disturbances were supported by frontal behavioral inventory (36), Columbia university scale for psychopathology in Alzheimer's disease (CUSPAD) and, interview with the participants and their informants by a psychiatrist.

### 2.3. MRI Study

Images from patients were obtained using a Philips Achieva 3.0 T scanner with a 16-channel SENSE coil. As part of a standardized protocol, all subjects had an single shot echo DTI pulse sequence with 32 diffusion encoding steps and two non-diffusion weighted images: *B*_0_ = 1000, repetition time [*TR*] = 7375 ms, echo time [*TE*] = 83.3 ms, flip angle = 90°, matrix = 128 * 128, slice thickness = 2 mm. Additionally, an anatomical and 3D T1-weighted was done with repetition time [*TR*] = 7.76, echo time [*TE*] = 3.72 ms, flip angle = 8°, matrix = 220 * 200, with slice thickness = 0.5 mm.

### 2.4. DTI Processing and Whole Tractography

**Figure 1:**
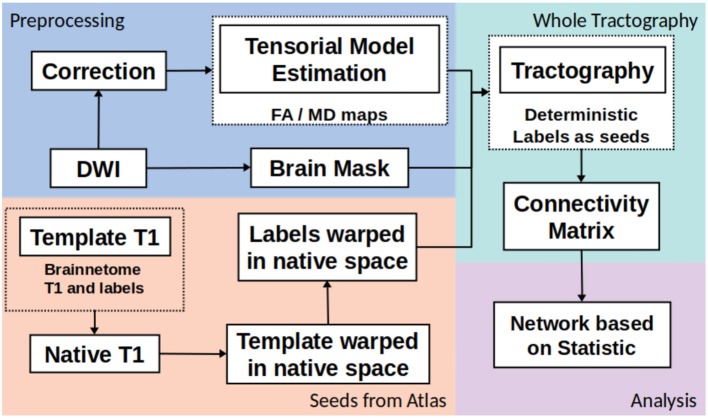
General pipeline of DTI processing and matrix construction.

The whole structural connectivity was obtained according to pipeline in the Figure 1. The preprocessing was directed to correct the images and estimated of diffusion orientation. The diffusion weighted images were affine-aligned to the first volume *B*_0_ to correct head motion. In addition, the eddy current distortion was corrected using the Eddy-Tool within the FMRI Software Library (FSL v4.1.5, http://www.fmrib.ox.ac.uk/fsl) (37). A mask brain was extracted using the BET tool to restrict all analysis (38). The diffusion tensor model was fitted with CAMINO (39). To fit models of the spin-displacement density function, a weighted linear fitting with the option RESTORE was used (40). This option allows for fitting the diffusion tensor in a robust way in the presence of outliers. The tensor eigenvalues (λ1, λ2, λ3), mean diffusivity ((λ1 + λ2 + λ3)/3), and fractional anisotropy (FA) were estimated at each voxel. The next step was creating the connectivity matrices based on FA and MD metrics with the Brainnetome Atlas with 246 labels (41). To adapt the labels from the atlas in the MNI space and the subject/(native) space, the labeled regions from the atlas were transformed to native space by a nonlinear symmetric registration with ANTs (42, 43) between the T1 image from the atlas and the T1 image at each subject. The matrix of transformation was used to register the labels from the atlas space to the native space.

The third step was the whole tractography estimation based on information from tensorial model, labels and a brain mask. A deterministic tractography (44) was done, the parameters were: threshold curve = 70, step-size with Euler tracker = 0.5, and anisotropy threshold = 0.01. The tracking algorithm was euler which is an extension of FACT proposed in by Mori et al. (45), with euler the tracking proceeds using a fixed step size along the local fiber orientation. With nearest-neighbor interpolation, this method may be very similar to FACT, except that the step size is fixed, whereas FACT steps extend to the boundary of the next voxel (distance variable depending on the entry and exit points to the voxel) (39). Each label from Brainnetome atlas in native space was a seed to tracking algorithm. The construction of connectivity-weighted matrices was made using the CONMAT tool from CAMINO (39). CONMAT takes as input a target image containing labeled regions (Atlas in native space), and streamlines output by previous step. It generates a matrix counting the number of streamlines connecting each pair of labels in the target image (39). In order to create a graph representation CONMAT was used to compute scalar statistics along the streamlines, such as the average FA and MD of each line connecting a pair of nodes. In the connectivity matrix, the labels represented the nodes and the edges were the average of FA or MD among the streamlines. A complete description to build the connectivity matrices could be consulted in http://camino.cs.ucl.ac.uk/index.php?n=Tutorials.ConnectivityMatrices.

### 2.5. Network Analysis

We used, in our analysis, network-based statistics (NBS) (46) to show differences among groups. NBS is a nonparametric statistical method to deal with the multiple comparisons problem on a graph. The method is used to control the family-wise error rate (FWER). The NBS is the graph analog of cluster-based statistical methods used in mass univariate testing on all pixels in an image. Rather than clustering in physical space, the NBS clusters in topological space where the most basic equivalent of a cluster is a graph component. FWER-corrected *p*-values are calculated for each component using permutation testing. NBS was used with the connectivity-weighted matrices of FA and MD in a deterministic and probabilistic way. The analysis of connectivity was directed to detect differences among groups in all cases. The significance level was *p* = 0.05 and 8000 permutations were done. Visualization results of structural connectivity networks were done with BrainNet viewer (47).

### 2.6. Gray Matter Analysis

Structural data were analyzed with FSL-VBM (48), an optimized VBM protocol (49) carried out with FSL tools (50). First, structural images were brain-extracted and gray matter-segmented before being registered to the MNI 152 standard space using non-linear registration (51). The resulting images were averaged and flipped along the x-axis to create a left-right symmetric, study-specific gray matter template. Second, all native gray matter images were non-linearly registered to this study-specific template and “modulated” to correct for local expansion (or contraction) owing to the non-linear component of the spatial transformation. The modulated gray matter images were then smoothed with an isotropic Gaussian kernel with a sigma of 3 mm. Finally, voxel-wise GLM was applied using permutation-based non-parametric testing (8000 permutations), correcting for multiple comparisons across space, and TFCE-based tests with randomize were used (52). TFCE is a cluster-based thresholding method which does not require an arbitrary cluster forming threshold. It take raw statistics image and producing an output image in which the voxel-wise values represent the amount of cluster-like local spatial support (53).

## 3. Results

The clinical differences between all groups are reported in the section on behavioral data and, in the sections that follow, the comparisons between variants of FTD based on FA and MD, specifically the networks with significant differences. Finally, the differences in GM among groups will be reported.

### 3.1. Behavioral Data

There was no significant effect of diagnosis on gender, age, disease duration, and education. According to results from a one-way ANOVA test and subsequent *post-hoc* analyses (Tukey's HSD) (Table 1), the global cognition measured by the MoCA test showed higher scores at bvFTD than svPPA and nfvPPA (*p* < 0.05 in both cases). Similar results were reported with some measures of language such as semantic fluency (*p* < 0.05) and naming (*p* < 0.05). In addition, the verbal comprehension evaluated by proverbs showed lower scores in patients with svPPA with respect to bvFTD. Differences between PPA variants were found with the spontaneous speech test at the repetition and pause rate (*p* < 0.05 in both cases). Behavioral symptoms evaluated by FBI were found in similar quantity at svPPA, nfvPPA and, bvFTD (*p* > 0.05). Additionally, psychopatological symptoms measured by CUSPAD were reported in all FTD variants and there were not significantly differences among them (*p* > 0.05).

### 3.2. PPA Variants vs. Controls

**Figure 2:**
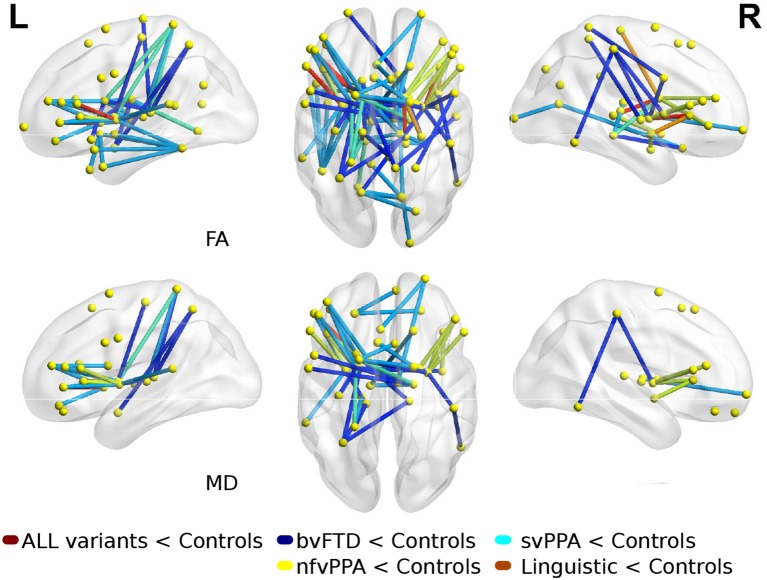
Atrophic networks in linguistic variants and bvFTD with respect to controls. Top figures show changes in fractional anisotropy (FA) and bottom show changes in mean diffusivity (MD). The figure shows alterations overlaid on a 3D template in the Montreal Neurological Institute's standard space in neurological convention.

Comparisons between FTD variants and controls showed different kind of atrophy patterns (see Figure 2). svPPA group had changes in 43 connections with lower values in FA, also 6 connections had higer values in MD. The main nodes altered were located in A37vl-R (ventrolateral area, inferior temporal gyrus), dCA-R (dorsal caudate), A7pc-R (lateral area, superior parietal lobule), dlPu-R (dorsolateral putamen) and, vmPu-R (ventromedial putamen). Furthermore, in nfvPPA the statistical analysis showed dCA-L (dorsal caudate), Otha-L (occipital thalamus), A44op-L (opercular area 44), dlPu (dorso lateral puramen)-L and, IFS-L (inferior frontal sulcus).

### 3.3. PPA Variants vs. bvFTD

**Figure 3:**
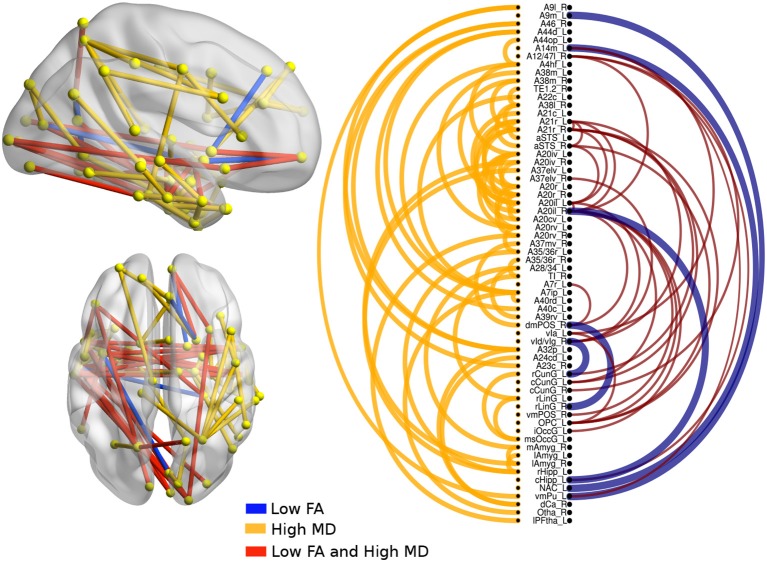
Atrophic networks in the svPPA variant with respect to bvFTD. The labels from Brainnetome represent the nodes and the edges show the changes in FA and MD. Only the edges with changes are shown. The whole list of the nodes with the names is located in Supplementary Data. The left of the figure shows alterations overlaid on a 3D template in the Montreal Neurological Institute's standard space in neurological convention. The right side shows the same results displayed in 2D with the names of nodes in the Brainnetome Atlas and displayed at *p* < 0.05.

**Figure 4:**
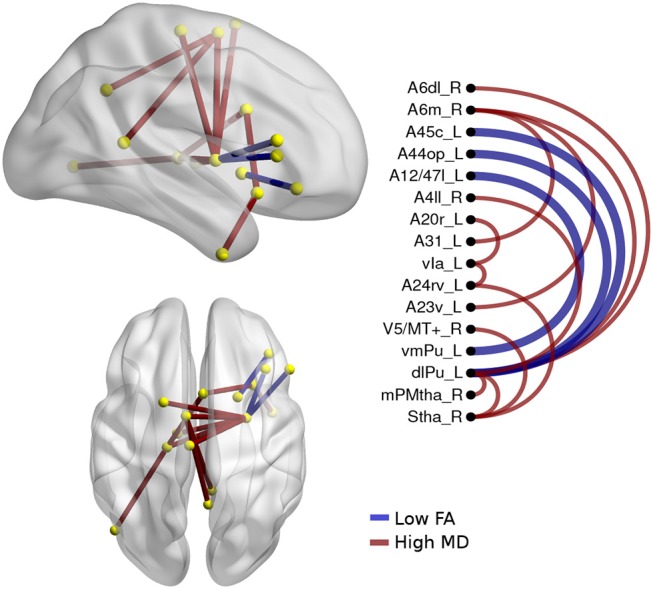
Atrophic networks in the nfvPPA variant regarding bvFTD, the labels from Brainnetome represents the nodes, and the edges show the changes in FA and MD. Only the edges with changes are shown. The whole list of the nodes with the names is located in Supplementary Data. The left side of the figure shows alterations overlaid on a 3D template in the Montreal Neurological Institute's standard space in neurological convention. The right side shows the same results displayed in 2D with the names of nodes in the Brainnetome Atlas and displayed at *p*<0.05.

There were significant differences between bvFTD and both PPA variants (see Figures 3, 4). First, 64 connections had changes in FA, MD, or both in the svPPA group. Additionally, the alterations were found in both hemispheres. Second, 16 connections had changes in nfvPPA and most of the nodes were located in the left hemisphere.

In svPPA patients (see Figure 3), high values in MD and low values in FA were found in the edges from the nodes in the left hemisphere: A20il (area intermediate lateral in temporal), A21r (area rostral in temporal), A12 (area lateral in orbital gyrus), aSTS (anterior superior temporal sulcus), and vIg (ventral insula). In addition, in the right hemisphere, the nodes with more changes were A21r (area rostral in superior temporal), A20il (area lateral in orbital gyrus), OPC (occipital polar cortex), and vI (ventral insula). Not only were changes in connectivity with low FA and high MD found, but there were changes in just one metric, either low FA or high MD. Nodes with at least three connections altered were A4hf (precentral gyrus head and face), A38l (superior temporal gyrus lateral area), A21r (area rostral in temporal), A20iv (area insular ventral), A20rv (area rostral of insula), A7ip (area intraparietal of superior parietal lobe), A32p (area pregenual in cingulate), and rHipp (rostral hippocampus).

The differences between nfvPPA and bvFTD (see Figure 4) were mainly in the nodes A6m.r (medial area from the superior frontal gyrus right) dlPut.l (dorsolateral putamen left), sThal.R (sensory thalamus right), vla.L (ventro agranular insula left), A24rv.L (rostro ventral area from the cingulate gyrus left), which presented high values in mean diffusivity. The connections between putamen left with A45c.L (caudal area in the inferior frontal gyrus left), A44op.L (opercular area left), and A12.L (orbital area left) showed high values in fractional anisotropy compared to bvFTD.

### 3.4. Comparisons Between PPA Groups

**Figure 5:**
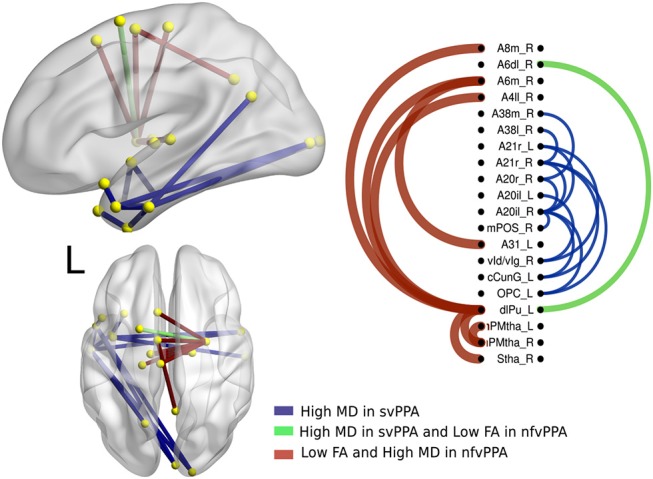
Atrophic networks in PPA variants. The labels from Brainnetome represent the nodes and the edges show the changes in FA and MD, only the edges with changes are shown. The whole list of the nodes with the names is located in Supplementary Data. The left side of the figure shows alterations overlaid on a 3D template in the Montreal Neurological Institute's standard space in neurological convention. The right side shows the same results displayed in 2D with the names of nodes in the Brainnetome Atlas and displayed at *p* < 0.05.

The alterations in structural connectivity in fractional anisotropy and mean diffusivity are shown in Figure 5, with a deterministic approach. Additionally, in these results, the differences between svPPA and nfvPPA were caused by the last group. There were no differences with the probabilistic approach in any measure.

A secondary analysis in linguistics variants shows specific differences between them. Hence, nodes disrupted in svPPA by a high value in MD were A20il.L (intermediate lateral area from inferior temporal left), A21r.L (rostral area in middle temporal left), A21r.R (rostral area in middle temporal right), vld.R (ventral dysgranular insula right), cCunG.L (caudal cuneus gyrus in occipital lobe left), and OPC.L (occipital polar cortex left). Additionally, the connection between dlPU.L (dorsolateral putamen left) and A4il.R (area 4 in paracentral lobe right), A6m.R (medial area 6 in superior frontal gyrus right), and A8m.R (medial area 8 in superior frontal gyrus right) was disrupted.

### 3.5. Gray Matter Analysis

**Figure 6:**
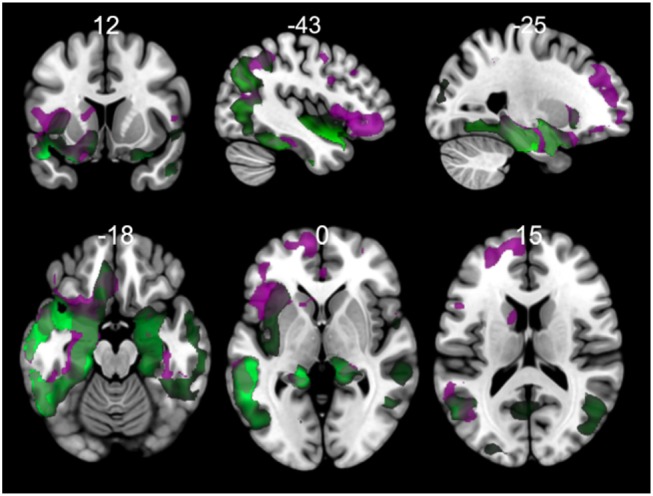
Comparisons of gray matter between controls and primary progressive variants. Each tile shows significant regions (correction by threshold-free cluster enhancement, *p* < 0.05, permutations: 8000) VBM analysis. Green: gray matter atrophy in svPPA, Purple: gray matter atrophy in nfvPPA. The table of main locations and their labels and coordinates can be found in Supplementary Data (Tables S1, S2).

The results of morphometry based on voxels shown gray matter changes in both PPA variants with respect to controls (Figure 6, Tables S1, S2 in Supplementary Data). svPPA patients had degeneration on both temporal lobes with a major extension in the left side. Other regions with changes insular cortex bilaterally, occipital fusiform left, occipital pole. The atrophy pattern in nfvPPA was found in insular cortex left, cingulate left, frontal opercular regions left, middle frontal bilaterally.

**Figure 7:**
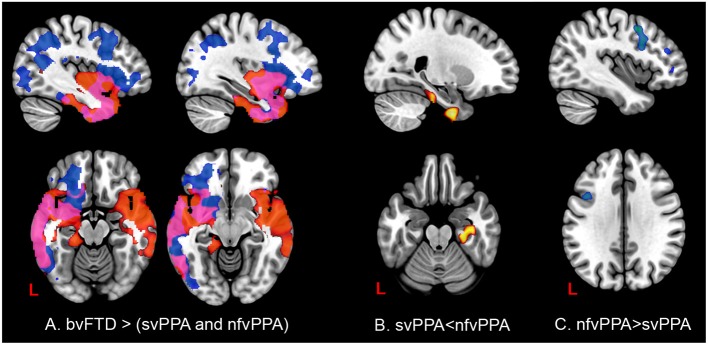
Comparisons of gray matter between behavioral variants and primary progressive variants. Each tile shows significant regions (correction by threshold-free cluster enhancement, *p* < 0.05, permutations: 8000) VBM analysis. **(A)** Regions with atrophy at linguistic variants regarding bvFTD. Blue: gray matter with changes in nfvPPa; Red: gray matter with changes in svPPA; Pink: gray matter with changes in svPPA and nfvPPA. **(B)** Gray matter changes in svPPA regarding nfvPPA. **(C)** Gray matter changes in nfvPPA regarding svPPA. The table of main locations and their labels and coordinates can be found in Supplementary Data.

In patients with svPPA compared with bvFTD had extended atrophy in the temporal bilateral area, which includes the temporal pole and the inferior, middle, and superior gyrus (Figure 7, Tables S3–S6 in Supplementary Data). Other regions affected were in posterior areas in the right hemisphere, such as the occipital cortex and the lingual and angular gyrus. In contrast, the atrophy shown in nfvPPA in comparison with bvFTD was found predominantly in the left hemisphere in the insular, frontal inferior, cingulate, and precuneus cortex areas. The differences between linguistic variants were located in svPPA in the inferior temporal and parahippocampal gyrus (left hemisphere). Conversely, nfvPPA patients had atrophy in the temporal pole, inferior frontal and middle frontal gyrus, at the left hemisphere.

## 4. Discussion

In the present study, a global tractography based on FA and MD to create structural connectivity in the three clinical variants of FTD was used. The analysis of structural networks showed distinct patterns in linguistic variants concerning bvFTD and between them. Patients with svPPA had a widely altered network from the temporal and occipital lobes and some specific nodes in the parietal and frontal inferior. The compromise of the ventral network in the temporal lobes was found bilaterally, with a major predominance in the left hemisphere. Additionally, the patients with nfvPPA showed a network in the frontal inferior regions, the premotor and motor cortex, and the basal ganglia. Further, the cortical regions atrophied were consistent with the subcortical networks altered in MD and FA. These findings provide evidence that a global connectivity with FA and MD, in conjunction with the Brainnetome Atlas, might be helpful in detecting cerebral signatures of network disintegration in PPA variants. We discuss these findings in relation to previous knowledge of the dual stream model for the language and neural bases of neurodegeneration in FTD.

Previous studies of PPA exposed in Table 2 have reported changes in white matter according to main subcortical fibers as uncinate, inferior longitudinal fasciculi, superior longitudinal fasciculi, corpus callosum and fornix. Common approach to study changes in PPA was tract based spatial statistics (TBSS). Despite of interest in specific tracts altered by dementia, TBSS is not tract-specific (66). The reduction of information by skeletonization could causes that different bundles to collapse on top of each other (66). Other techniques reported by Elahi et al. (59) with DTI-TK, Grossman et al. (18) with Pipedream, and Tetzloff et al. (65) with ANTs could be contribute to extend the knowledge about tracks altered by PPA. The present study based on whole tractography approach as well as an incremental of granularity could be useful to capture short cortico-cortical connections in an spatial map.

**Table 2:** Comparison of fractional anisotropy and mean diffusivity changes in DTI studies of PPA.

**References**	**Sample**	**Method**	**Uncinate**	**ILF**	**SLF**	**CC**	**Fornix**	**Other**
Acosta-Cabronero et al. (54)	10 svPPA	TBSS	B	B	B	nt	nt	
Agosta et al. (55)	5 svPPA	Fiber tracking	L	L	L	X	nt	
Botha et al. (12)	40 PPA,	FA and MD maps	nt	B	nt	X	nt	
	12 agramatic		L	nt	B	X	nt	
	9 svPPA,		B	B	nt	nt	nt	L. cingulum
	52 lvPPA		nt	B	B	nt	nt	
Bouchard et al. (56)	10 svPPA	TBSS	B	nt	L	X	X	C. cingulum
de Oliveira et al. (57)	4 PPA	Fiber tracking	nt	L	L	nt	nt	
Downey et al. (58)	15 svPPA	TBSS	B	R	nt	X	nt	B. cingulum
Elahi et al. (59)	19 svPPA	DTI-TK, TBSS	nt	nt	nt	X	X	
	14 nfvPPA		nt	nt	nt	nt	X	Internal capsule
Grossman et al. (18)	15 nfvPPA	Pipedream, FA maps	L	B	L	X	X	
Laccarino et al. (60)	10 svPPA	Ptr	B	B	nt	nt	nt	
Mahoney et al. (61)	33 nfvPPA	TBSS	B	B	L	nt	X	
	10 lvPPA		L	L	nt	nt	X	Cortico-spinal
	10 svPPA		B	L	nt	nt	nt	B. cingulum
Marcotte et al. (62)	13 nfvPPA	FA and MD maps, Ptr	nt	nt	L	nt	nt	
	12 svPPA		B	B	nt	nt	nt	
Nguyen et al. (63)	16 svPPA	TBSS	nt	nt	nt	nt	nt	R. Insula
	16 nfvPPA		nt	nt	nt	X	nt	B Insula
Schwindt et al. (64)	9 nfvPPA	TBSS	B	B	B	X	nt	
	9 svPPA,		B	B	B	X	nt	
Tetzloff et al. (65)	11 aPPA	ANTs	nt	R	B	nt	nt	L external capsule
	20 PPAOS		nt	nt	B	nt	nt	Internal capsule

*TBSS, tract based spatial statistics; PTr, Probabilistic tractography; ANTs, Advanced Normalization Tools; L, Left; R, Right; X, without laterality; B, both sides; nt, no tested*.

Studies of PPA have shown not only language deficits but also low performances in emotion recognition and behavioral disturbances (67). For instance, patients with svPPA have shown more behavioral disturbances than other variants as lvPPA and nfvPPA (68). Our analysis based on FBI and CUSPAD scores showed similar disturbances and psychiatric symptoms between bvFTD and PPA variants. Due to presence of behavioral disturbances our analysis included contrasts between bvFTD and PPA variants as well PPA and controls.

With respect to language models, which could supports the alterations in PPA the dual model presents two streams: the lexical-conceptual and the motor stream (69). This dual-stream model is supported first by the ventral stream from the posterior middle and inferior temporal gyrus to the anterior middle temporal gyrus, which support auditory comprehension. Additionally, a recent study showed that tracts, such as the uncinate fasciculus, the superior longitudinal fasciculus, the retrolenticular part of internal capsule, the inferior frontooccipital fasciculus, posterior thalamic radiation, and posterior corona radiata are associated with this stream (70). Similarly, other studies supported alterations in white matter in uncinate (18, 54, 56), inferior longitudinal fasciculi (55, 57, 61, 71) Patients with svPPA have impaired comprehension of content words (nouns, verbs, adjectives), anomia, and semantic impairment (4, 72).

In this study, the svPPA group had low values of FA and high values of MD in connections from the intermediate area in the anterior temporal lobe to the rostral area, the pole of the occipital cortex, and the caudal cuneus, which are associated to inferior longitudinal fasciculus. Nevertheless, the networks altered in this study not only included the left hemisphere but also the right hemisphere. The atrophy on the right side has been associated with the recognition of familiar faces and voices (73), emotional withdrawal, and irritability (23). Similarly, another report showed the importance of this area to mentalizing abilities and empathy in patients with FTD (74). Accordingly with gray matter analysis in our study svPPA patients had a bilateral atrophy in temporal regions with more left sided involvement in comparison with bvFTD and controls. Bilateral atrophy in svPPA has been reported previously both gray and white matter analysis (25, 56). Additionally, both hemispheres underpinned the semantic and conceptual representations (75), which are affected in svPPA.

The second aspect of the dual model is the dorsal stream, which is related to phonological-motor aspects of speech production (69, 70). This stream might extend from anterior speech areas in the frontal (pars opercularis, premotor areas) to posterior areas of the supramarginal gyrus (70). Patients with nvfPPA showed cortical atrophy in the opercular area and the rostral area in the left inferior frontal lobe. In connectivity terms, both areas showed connections altered in the left putamen. A recent study has shown the importance of connectivity from putamen in both hemispheres to grammatical processing and broader semantic processes (76). Grammar production and comprehension is an important part of changes in language in nfvPPA patients (7). Atrophy in basal ganglia and bulbar stream might be found in patients with nfvPPA owing to other common pathologies associated with nfvPPA, such as cortico-basal degeneration and supra-nuclear palsy (26).

The parcellation cortex has been a factor that can affect the metrics used to research brain networks (77). Owing to this, in this study, the atlas selected to create the global tractography was the Brainnetome Atlas. This atlas contains 246 subregions of the bilateral hemispheres and is based on connectivity architecture. Compared to the AAL atlas with 90 regions, the high number of regions in the Brainnetome Atlas can be useful to detect with more accuracy alteration in networks both small and large. This granularity allows for identifying small networks altered, for instance, between rostral and ventral areas in the temporal pole in svPPA. A secondary aspect in this study was the estimation of both FA and MD. Both metrics allowed for identifying more complete alterations in the networks, as there were differences in the networks depending on the metric used and sometimes the same networks showed high values in MD and low values in FA. This is an argument to highlight the use of both metrics in a clinical analysis in DTI.

### 4.1. Limitations

This study has several limitations. The sample size was relatively small, consisting of patients with svPPA and nfvPPA, owing to the careful matching of three groups. In addition, assessments were cross-sectional without pathological confirmation, and the severity stage was moderate. However, our sample size was similar to other studies (17, 61, 78, 79). All patients (svPPA, nfvPPA, and bvFTD) showed behavioral disturbances and similar scores in CUSPAD and FBI due to disease duration (5 years and more). The consequence of that was patients in moderate stages. Nevertheless, the first consultation did not always occur in mild stages, as language changes are not familiar symptoms, such as memory in Alzheimer's disease. Due to presence of behavioral disturbances the clinicians of memory clinic made the diagnosis by consensus. This consensus was supported by an evaluation by a psychiatrist expert in FTD and the use of Rascovsky criteria (28). At least four patients were diagnosed previously by another center with Lund Manchester criteria (27). However theses patients were re-evaluated by the clinic memory according with Rascovsky criteria (28).

### 4.2. Conclusions

In the present study, we showed that nfvPPA and svPPA have a distinct pattern of atrophy in networks based on MD and FA. This pattern might be useful to explain the decline in speech and language, as well as the occurrence of disturbances in behavior. For instance, the deep alteration in networks related to the ventral stream might be associated with changes in language in svPPA. In contrast, patients with nfvPPA showed changes in the dorsal stream. The global and structural connectivity might be useful to understand, at the same time, the language and behavioral disturbances. These findings generated by a global tractography from small regions can be useful to contrast the cognitive model of speech/language and their changes in neurodegenerative diseases.

### 4.3. Tables

The Tables S3–S6 show regions with cortical atrophy according to each t-test contrast with VBM analysis.

## Data Availability

The datasets for this manuscript are not publicly available because The raw data from patients are only available in the institutional repositories. Requests to access the datasets should be directed to dianamat@javeriana.edu.co.

## Ethics Statement

All participants and tutors provided written informed consent in agreement with the Helsinki declaration. The protocol was approved by the Hospital Universitario San Ignacio/Pontificia Universidad Javeriana committee.

## Author Contributions

PR and AR designed the study and implementation of the research. PR contributed to the analysis of the results and to the writing of the manuscript. DM and FU performed a critical revision of the article.

### Conflict of Interest Statement

The authors declare that the research was conducted in the absence of any commercial or financial relationships that could be construed as a potential conflict of interest.
