# Supplementary material for: Networks Disrupted in Linguistic Variants of Frontotemporal Dementia
Source: Front Neurol. 2019 Aug 23;10:903. doi: 10.3389/fneur.2019.00903 (PMC6716200; doi:10.3389/fneur.2019.00903)
Supplement: Supplementary file 1 [file Table_1.pdf]

Supplementary Data

The next tables show regions with cortical atrophy according to each t-test contrast with VBM analysis.

Table S1: Peak Coordinates from VBM results in MNI space, Contrast Controls>svPPA

Index	MAX	x	y	z	Oxford atlas labels	Brainnetome
15878	>0.999	-62	-20	-22	Insular Cortex	A21c_L
11126	>0.999	34	10	-26	Temporal Fusiform	A38m_R
288	>0.999	50	-38	22	Parietal Operculum Cortex	A40rv_R
148	>0.999	46	-84	-14	Lateral Occipital Cortex inferior division	iOccG_r
141	>0.999	-30	-60	-8	Occipital Fusiform Gyrus	a37m_L
139	>0.999	14	-84	10	Occipital Pole	cCunG_R
134	>0.999	44	-58	26	Lateral Occipital Cortex superior division	A39rv_R
122	>0.999	46	-68	8	Lateral Occipital Cortex inferior division	V5/MT+_R
101	>0.999	-18	-38	46	Postcentral Gyrus s	A1/2
100	>0.999	28	-68	-6	Occipital Fusiform Gyrus	a37mv_R

Note: MAX = maximum intensity within the cluster, maximum probability $(1 - p)$

Table S2: Peak Coordinates from VBM results in MNI space, Contrast Controls>nfvPPA

Voxels	MAX	x	y	z	Oxford atlas labels	Brainnetome
11039	1	-28	28	-4	Insular Cortex	A12/47L
998	1	32	-2	-22	Parahippocampal Gyrus	lAmyg_R
668	1	-4	-58	34	Precuneous Cortex	A31_L
650	1	-10	20	36	Cingulate Gyrus	A9m_L
642	1	34	22	4	Frontal Operculum	dla_R
598	1	-16	-42	48	Cingulate Gyrus	A5m_L
329	1	-26	46	18	Frontal Pole	A9/46d_L
323	1	-24	-4	48	Middle Frontal	A6vl_L
181	1	48	-52	24	Lateral Occipital	A39rv_R
173	1	-50	-34	20	Parietal Operculum	A40rv_L
137	1	56	0	8	Central Opercular	A4tl_R
137	1	34	-2	50	Middle Frontal	A6vl_R

Note: MAX = maximum intensity within the cluster, maximum probability $(1 - p)$

Table S3: Peak Coordinates from VBM results in MNI space, Contrast svPPA>bvFTD

Voxels	MAX	x	y	z	Oxford atlas labels	Brainnetome labels
34223	1	-54	-2	-36	Superior Temporal Gyrus	A44d_L
271	0.969	38	-78	-14	Lateral Occipital Cortex	OccG_R
199	0.964	24	-70	2	Intracalcarine Cortex	vmPOS_R
198	0.956	46	-66	0	Lateral Occipital Cortex	V5/MT+_R
119	0.953	-34	-68	20	Angular Gyrus	A39rv_L
69	0.965	-16	36	52	Superior Frontal Gyrus	A8dl_L
64	0.961	56	-38	24	Supramarginal Gyrus	A40c_R
16	0.953	28	-92	-28	Lateral Occipital Cortex	
9	0.952	16	-82	-10	Lingual Gyrus	cLinG_R
2	0.95	-44	-88	-2	Lateral Occipital Cortex	iOccG_L

Note: MAX = maximum intensity within the cluster, maximum probability $(1 - p)$

Table S4: Peak Coordinates from VBM results in MNI space, Contrast nfvPPA>bvFTD

Voxels	MAX	x	y	z	Oxford atlas labels	Brainnetome labels
33464	1	-46	2	-44	Insular Cortex	dId_L
7594	0.991	40	22	-8	Insular Cortex	vId/vIg_R
271	0.961	44	-60	38	Lateral Occipital Cortex	A39rv_R
235	0.962	-6	-54	30	Cingulate Gyrus	A23d_L
143	0.957	20	56	36	Frontal Pole	A9/46d_R
81	0.959	-58	-22	50	Postcentral Gyrus	A1/2/3ulhf_L
27	0.955	-4	-66	58	Precuneous Cortex	A7r_L
15	0.952	50	-52	24	Angular Gyrus	A40c_R
8	0.952	14	-56	34	Precuneous Cortex	A7m_R
3	0.95	46	-30	-8	Middle Temporal Gyrus	A21c_R

Note: MAX = maximum intensity within the cluster, maximum probability $(1-p)$

Table S5: Peak Coordinates from VBM results in MNI space, Contrast svPPA>nfvPPA

Voxels	MAX	x	y	z	Oxford atlas labels	Brainnetome labels
345	0.983	-38	6	48	Middle Frontal Gyrus	IFJ_L
272	0.972	-2	-66	46	Precuneous Cortex	A31_L
42	0.955	-32	50	14	Frontal Pole	A46_L
40	0.959	-38	40	22	Frontal Pole	A9/46v_L
22	0.954	-42	44	-2	Frontal Pole	A45r_L
20	0.954	-46	24	4	Inferior Frontal Gyrus	A44op_L
6	0.951	-46	42	12	Frontal Pole	A9/46v_L

Note: MAX = maximum intensity within the cluster, maximum probability $(1-p)$

Table S6: Peak Coordinates from VBM results in MNI space, Contrast nfvPPa>svPPA

Voxels	MAX	x	y	z	Oxford atlas labels	Brainnetome labels
705	0.996	20	-2	-44	Temporal Fusiform Cortex	A38m_R
605	0.991	28	-28	-28	Parahippocampal Gyrus	TL_R
111	0.963	-20	-10	-44	Temporal Fusiform Cortex	A20rv_L

Note: MAX = maximum intensity within the cluster, maximum probability $(1-p)$
